# Artificial Intelligence Applied to a First Screening of Naevoid Melanoma: A New Use of Fast Random Forest Algorithm in Dermatopathology

**DOI:** 10.3390/curroncol30070452

**Published:** 2023-06-23

**Authors:** Gerardo Cazzato, Alessandro Massaro, Anna Colagrande, Irma Trilli, Giuseppe Ingravallo, Nadia Casatta, Carmelo Lupo, Andrea Ronchi, Renato Franco, Eugenio Maiorano, Angelo Vacca

**Affiliations:** 1 Section of Pathology, Department of Precision and Regenerative Medicine and Ionian Area (DiMePRe-J), University of Bari “Aldo Moro”, 70124 Bari, Italy; anna.colagrande@gmail.com (A.C.); giuseppe.ingravallo@uniba.it (G.I.); eugenio.maiorano@uniba.it (E.M.); 2 LUM Enterprise srl, S.S. 100-Km.18, Parco il Baricentro, 70010 Bari, Italy; massaro@lum.it; 3 Department of Management, Finance and Technology, LUM—Libera Università Mediterranea “Giuseppe Degennaro”, S.S. 100-Km.18, Parco il Baricentro, 70010 Bari, Italy; 4 Odontomatostologic Clinic, Department of Innovative Technologies in Medicine and Dentistry, University of Chieti “G. D’Annunzio”, 66100 Chieti, Italy; trilliirma@gmail.com; 5 Innovation Department, Diapath S.p.A., Via Savoldini n.71, 24057 Martinengo, Italy; nadia.casatta@diapath.com (N.C.); carmelo.lupo@diapath.com (C.L.); 6 Pathology Unit, Department of Mental Health and Physic and Preventive Medicine, University of Campania “Luigi Vanvitelli”, 80138 Naples, Italy; andrea.ronchi@unicampania.it (A.R.); renato.franco@unicampania.it (R.F.); 7 Centro Interdisciplinare Ricerca Telemedicina—CITEL, Università degli Studi di Bari “Aldo Moro”, 70124 Bari, Italy; angelo.vacca@uniba.it

**Keywords:** fast random forest (FRF), algorithm, naevoid melanoma (NM), artificial intelligence (AI), dermatopathology, BPMN clinical processes

## Abstract

Malignant melanoma (MM) is the “great mime” of dermatopathology, and it can present such rare variants that even the most experienced pathologist might miss or misdiagnose them. Naevoid melanoma (NM), which accounts for about 1% of all MM cases, is a constant challenge, and when it is not diagnosed in a timely manner, it can even lead to death. In recent years, artificial intelligence has revolutionised much of what has been achieved in the biomedical field, and what once seemed distant is now almost incorporated into the diagnostic therapeutic flow chart. In this paper, we present the results of a machine learning approach that applies a fast random forest (FRF) algorithm to a cohort of naevoid melanomas in an attempt to understand if and how this approach could be incorporated into the business process modelling and notation (BPMN) approach. The FRF algorithm provides an innovative approach to formulating a clinical protocol oriented toward reducing the risk of NM misdiagnosis. The work provides the methodology to integrate FRF into a mapped clinical process.

## 1. Introduction

Malignant melanoma (MM) is known to be the great mimic of dermatopathology [1] as it can mimic different types of malignant neoplasms, not only from a histopathological/morphological point of view but also through potential aberrant expressions of immunohistochemical markers [2] that contribute to confusing the pathologist, making differential diagnosis even more difficult. With respect to MM, naevoid melanoma (NM) represents the most frequent cause of financial claims [3] in the field of MM diagnosis as it represents a poorly diagnosed variant of melanoma which simulates a naevus and can often escape even the most experienced dermatopathologist. Levene [4] initially reported NM in 1980 and assigned it the designation of “verrucous and pseudonaevoid melanoma” in doing so. The definition of “naevoid malignant melanoma” was first employed in 1985 by Schmoeckel et al. [5], who reported 33 patients with malignant melanomas that exhibited histological features resembling benign melanocytic lesions and underwent follow-ups for at least five years. NM most frequently involved the backs and limbs of male patients with a mean age of 57 years in a clinicopathological investigation of 20 cases carried out by Bleessing et al. [6]. Over 50% of cases had a clinical diagnosis of benign lesions, while only 10% had a histology diagnostic at first. Furthermore, a morphological evaluation of 20 patients with a minimum of three years of follow-up found that four tumours recurred and three metastasized, all with fatal outcomes. In this study, the average Breslow thickness was 2.5 mm, and there was no difference in prognosis between NM and conventional malignant melanoma [7]. Various authors have summarized the morphological criteria that are the most useful in making a correct diagnosis of NM, beginning with the mandated recommendation that any melanocytic lesion, particularly when NM is suspected, should be assessed not only at panoramic magnifications (×2, ×4) but also at higher magnifications (×10, ×20, ×40 etc.) in order to study and investigate the more subtle morphological features in the lesions [8]. Interestingly, in a 2017 paper, Cook, M.G., et al. [9] conducted a retrospective study of 89 NM cases that were divided into two groups consisting of 11 cases and 78 cases, respectively.

The first group (n = 11) consisted of clinically papillomatous lesions which, upon histology, consisted of predominantly intradermal melanocytes with hyperchromatic and angulated nuclei, numerous dermal mitoses, and no real maturation in depth; in this group of lesions, there was little or no junctional component.

The second group (n = 78) consisted of flat or shallow dome-shaped lesions and presented melanocytes with a morphological appearance that was referred to as that of a “maturing naevoid melanoma” with a greater possibility of the presence of a dysplastic junctional melanocytic component and, in addition, the presence of true melanocyte nests in the deep dermis. From all that we have said, it is quite clear that there are rather clear but also “subtle” criteria that must be followed precisely; otherwise, there is a risk of misdiagnosis and all that it would entail for the diagnostic–therapeutic and care pathways of the NM-affected patient.

Artificial intelligence (AI) has changed clinical and diagnostic routines decisively [10]. Suffice it to say that the application of AI algorithms to the medical field has undergone a surge in the last decade, with pathology also being affected quite extensively [11]. When discussing the application of AI to histopathological (and other) diagnostics, one must always divide the two types of AI into supervised machine learning, machine learning (ML) and deep learning (DL). More specifically, ML is a subset of AI that deals with creating systems that learn or improve performance based on the data they use; in this framework, validation (internal and/or external) is always required, and algorithms can be “supervised” (known outcome) or “unsupervised” (clustering) [12].

In this paper, after a previous work on MM [13], we present preliminary data on the training of an ML algorithm called fast random forest (FRF) which is applied to a dataset of histopathological images of NM; we discuss its strengths and limitations and provide a critical prospective for the near and distant future.

## 2. Materials and Methods

### 2.1. Data Acquisition

For the processes of training, validation and testing, we used a dataset of 18 photomicrographs of NM, originally taken at 1920 × 1088 pixels using a NanoZoomer S60 Digital slide scanner C13210-04 at diverse magnifications (from 4× to 20×). The images were obtained from patients with histologically confirmed diagnoses of NM in the period from January 2010 to December 2022. For each patient, demographic, clinical, histopathological and immunohistochemical features were recorded for routine pathological practice; moreover, informed consent was obtained from all patients, and the local ethical committee approved this study by CITEL (Dipartimento di Scienze Biomediche e Oncologia Umana—Dimo U.O.C. Medicina Interna “G. Baccelli” Policlinico di Bari, number 5732).

Haematoxylin and eosin (H&E) staining was used for histopathological analysis and for each case the diagnoses were reviewed in a double-blind manner by two experienced dermatopathologists (A.C. and G.C.). Subsequently, a dermatopathologist (G.C.) selected the most representative areas with the minimum criteria for a possible diagnosis of NM. The criteria used in the pre-selection phase are summarized in Table 1, and the clinical, histological and immunohistochemical features of the 18 cases analysed are reported in Table 2.

### 2.2. Algorithm 

**Figure 1:**
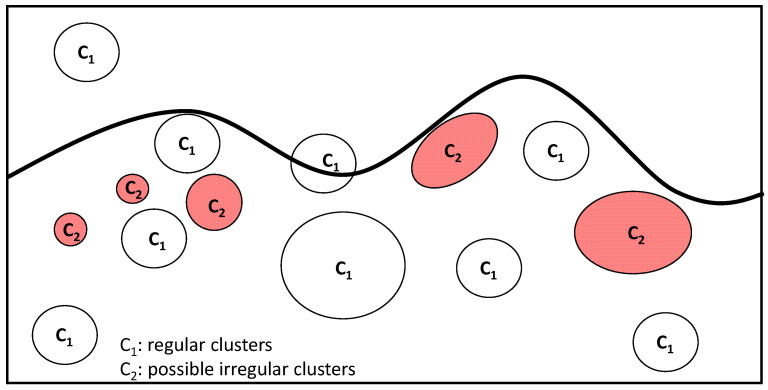
NM pre-screening training model approach based on the identification of a class of possible anomalous clusters and of a class of regular clusters. The FRF algorithm training was performed on the same image.

The algorithm applied for feature extraction was the fast random forest (FRF) algorithm, a powerful supervised machine learning algorithm that optimizes the performance of the random forest (RF) algorithm with respect to computational speed and classification accuracy: it defines the best decision tree split condition step by step, thus avoiding unnecessary computations, extracting clusters of pixels appertaining to similar classes [1,14,15]. Specifically, the FRF algorithm implements Weka libraries and has been primarily used in industrial applications [15] and successively applied to medical images [13]. Theoretically, a similar class is identified by a group of pixels with different greyscale intensities and specific distances. The FRF algorithm is trained by defining classes in the same image: the “ambiguous” areas are selected as classes to focus attention on, while the other areas are classified as “regular” classes. Only two main classes are identified in each image: the alerting class and the regular class; in this way, this classification approach provides probability maps concerning only regular and anomalous pixel distributions. The training was performed in each image by classifying different clusters highlighted by the dermatopathologist as part of the “anomalous” class. In Figure 1 is illustrated a scheme related to the training model applied to the medical images analysed (all images with a resolution of 1920 × 1088 pixels were acquired by a NanoZoomer S60 Digital slide scanner C13210-04, Hamamatsu, Japan). The advantage of the proposed approach is that it punctually extracts pixels with the same features as the appertaining class (the intensity of pixels with defined distances from the other pixels with a specific intensity) from the whole image. In Table 3, the difference between the classification approach used in our previous paper [13] and the one applied in this paper, which is suitable for an initial screening of NM, is detailed. The parameters estimated to quantify the FRF algorithm’s performance are recall and precision [16]. 

The performed analysis was enhanced via the colour distribution within the 3D RGB colour space, thus counting the number of pixels contained in each image (pixels with a high probability of classifying an NM region were highlighted in red). Specifically, the NM information (possible alerts for clusters to classify in the pre-screening process) is expressed in terms of the number of red pixels in equivalent areas expressed in mm^2^ and as a percentage (%). The procedure to estimate the number of red pixels is as follows:

- The probabilistic images of both the classes C_1_ and C_2_ are extracted;
- For each probabilistic C_2_ image, a filter enhancing the pixels with the highest values in red is applied by considering a red threshold of about 0.38 and a dark background;
- The RGB distributions of the filtered images, counting only the red pixels (which have higher probabilities of classifying NM), are plotted.

The equivalent area expressed as a percentage is a new probabilistic indicator measuring the potential classification of NM. The procedure followed to estimate the probabilistic indicator is as follows:

1. For each image, the number of pixels constituting the scale bar line (each image indicates a scale bar of 500 microns) is counted;
2. The length and the height of each image are estimated in microns;
3. The total area of each image is estimated in pixels^2^;
4. The total area of each image is estimated in microns^2^;
5. The red pixels of the processed images (indicating a high probability of NM) are counted;
6. The equivalent area of the red pixels is estimated and expressed in microns^2^;
7. The same area of the red pixels is expressed in mm^2^;
8. The percentage value (the ratio between the total image area in mm^2^ and the equivalent area of the red pixels in mm^2^) is expressed.

Table 4 lists the best FRF hyperparameters for optimising the image processing analysis.

### 2.3. Pre-Screening Clinical Process Modelling

The pre-screening clinical process based on the integration of the FRF analysis was designed by using the business process modelling and notation (BPMN) approach. The BPMN is an international standard (ISO/IEC 19510:2013 “Information technology, Object Management Group Business Process Model and Notation”) used to design processes, including machine learning decision-making tools [17], and it is adopted to improve healthcare organizational processes [18]. The goal of the adopted BPMN approach was the standardization of the new clinical pre-screening protocol to facilitate scientific approval. The BPMN models will be revised in another paper updating the protocols and refining the procedure to further decrease the NM risk. 

In detail, the clinical–pathological process was mapped following the seven consequent phases listed below:

1. Sections stained in haematoxylin and eosin (H&E) are digitally acquired by a scanner;
2. Different scanning modalities are tested to determine the best screening approach for monitoring different areas (for example, scanning in 20× and 40× mode (15 mm × 15 mm), 0.46 μm/pixel (20×) 0.23 μm/pixel resolution (40×); 20×: 1 mm scale bar; 40×: 500 micron scale bar);
3. The most significant slide from a single patient containing more diagnostic features (more significant images) is chosen;
4. Supervising the lesion and analysing the main characteristics to define the main clinical scenario, using the 2×–4× analysis as a “first look” to evaluate the more general characteristics of the lesion;
5. Conducting an FRF analysis of the selected digital slides (pre-screening);
6. Carrying out an FRF threshold check (image post-processing): if the 12% threshold is exceeded, the “suspected” case is looked at first and then the remaining cases are analysed (patient priority in the analysis approach); the threshold value can be changed in the function of the image filtering threshold and by assessing the real evolution of the NM in the future;
7. Once the suspected case of NM has been analysed, immunohistochemical investigations are carried out for Melan-A, HMB-45, p16 and PRAME.

## 3. Results

**Figure 2:**
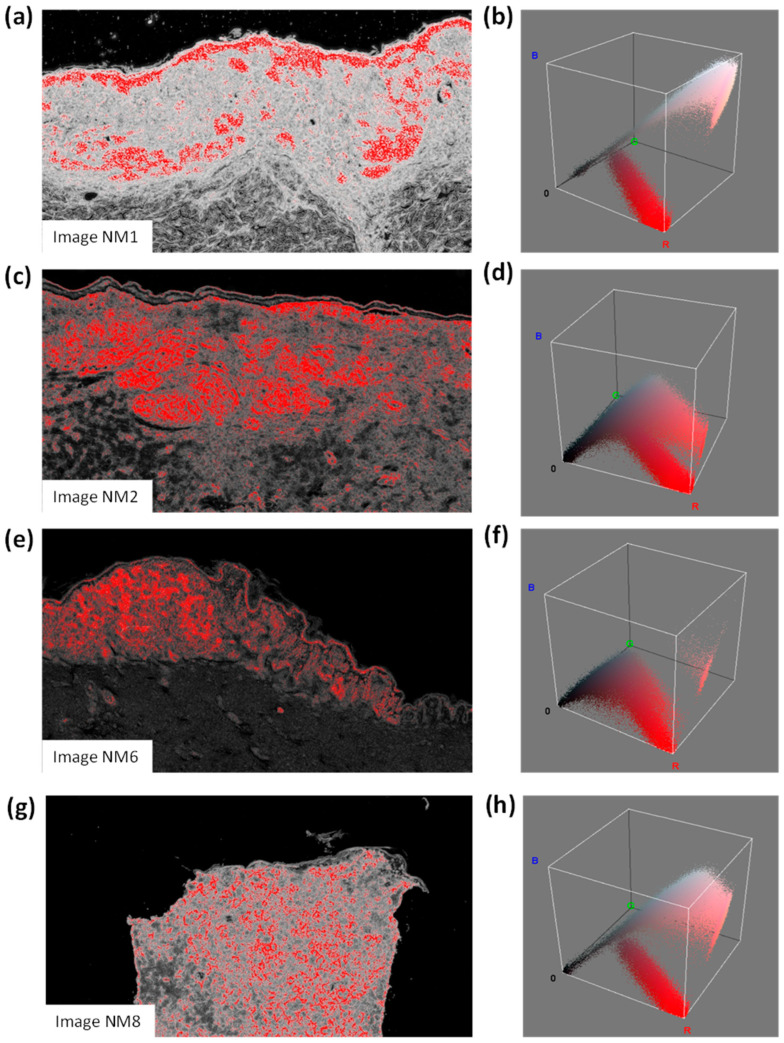
(**a**) Image NM1 and (**b**) related colour distribution within the 3D RGB colour space; (**c**) image NM2 and (**d**) related colour distribution within the 3D RGB colour space; (**e**) image NM6 and (**f**) related colour distribution within the 3D RGB colour space; (**g**) image NM8 and (**h**) related colour distribution within the 3D RGB colour space.

Image processing was performed in the self-learning FRF analysis, which focused on classifying possible areas of naevoid melanoma (NM). The FRF extracted the probabilistic images from the photomicrographs (an example in Figure 2), highlighting the region where NM is the most probable. This analysis was enhanced via the colour distribution within the 3D RGB colour space, thus estimating the number of pixels contained in each image (pixels with a high probability of classifying a NM region were highlighted in red). Table 5 lists the NM information in terms of the number of red pixels, equivalent areas expressed in mm^2^ and the percentage (see the last three columns) of four images in the pool of used pictures. The equivalent area expressed as a percentage could be a probabilistic indicator measuring the potential classification of NM.

**Figure 3:**
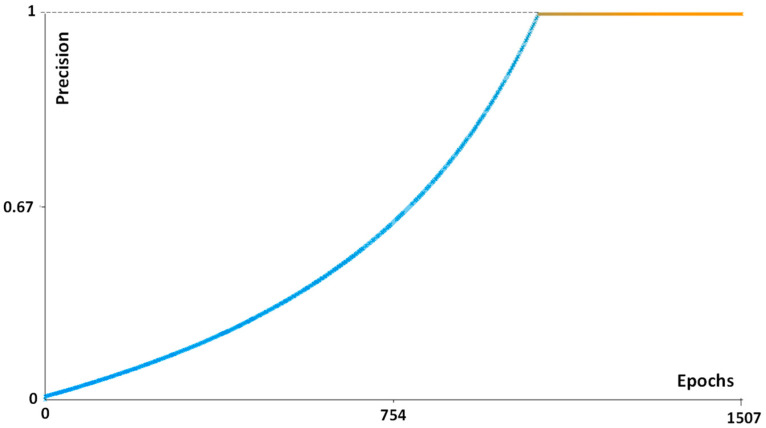
FRF Precision parameter confirming the good algorithm performance (NM2 case).

The good performance of the FRF algorithm is verified via the estimated Precision parameter in Figure 3. Similar *Precision* trends are found for all the images analysed. Other examples of performance indicators are discussed in Appendix A. 

**Figure 4:**
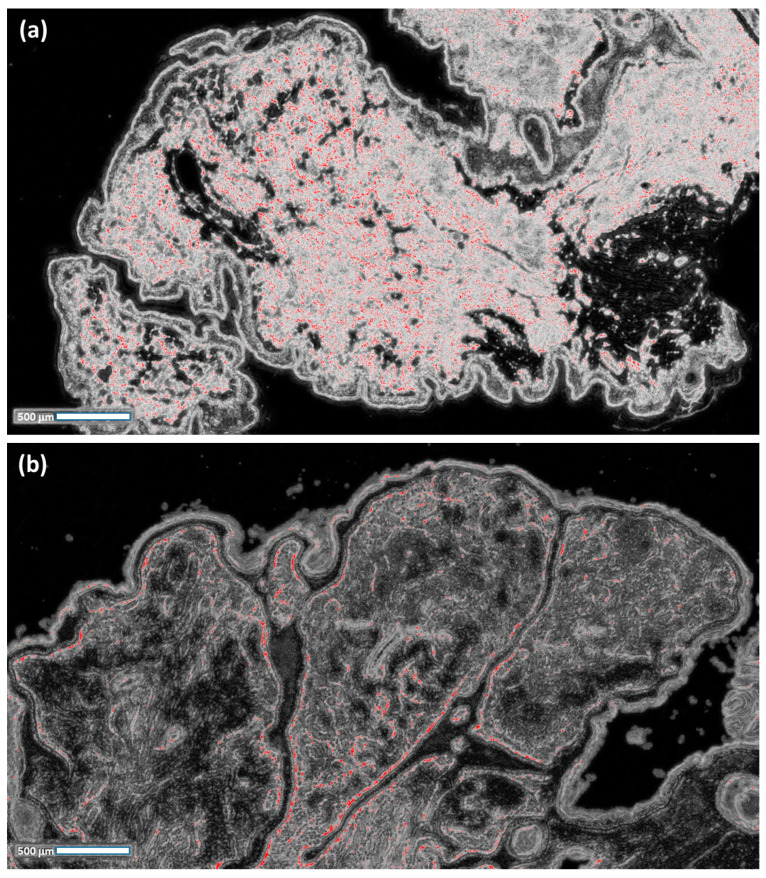
(**a**,**b**) Image processing of benign naevus cases, using the training model used for the naevoid melanoma cases (the threshold values are significantly less than 12%).

In order to explain the principal selection, we tested a further 18 cases of benign naevus (Unna naevus), which is a differential diagnosis with NM (Figure 4). The goal was to create a training model that is useful in detecting possible and probable areas of NM: for this purpose, the training model [19] was created by targeting significant pixel areas in NM cases. The execution of the trained FRF algorithm provides threshold values for the benign cases that are significantly less than 12%, thus proving the efficiency of the algorithm in better identifying dangerous cases. An image processing limit is part of the correct setting of the pixel intensity filter when selecting thresholds to estimate (a wrong setting could provide wrong results).

## 4. Discussion

Naevoid melanoma represents a very important challenge in dermatopathology and even today, its misdiagnosis represents one of the main dangers, even in an ultra-specialist setting. Over time, there have been several descriptions of this entity in which it is important to emphasise the differences in the authors’ perspectives regarding the presence of a junctional component. In more detail, some authors considered and restricted the use of the term “naevoid” only to those cases involving a lesion that resembled a benign naevus with a complete absence of a junctional component [20], but others [21] also included in their case histories cases in which an intraepidermal lentiginous component was recognisable. In recent years, artificial intelligence has shown that it can also assist in the field of dermatopathology, not only from a purely diagnostic perspective but also from educational, resource and screening perspectives [22,23,24]. In a very recent paper, Ibraheim, M. K. et al. [25] addressed these topics, emphasising, among other things, the importance of using artificial intelligence in dermatopathology as a screening tool, and our paper attempts to move precisely in this direction.

**Figure 5:**
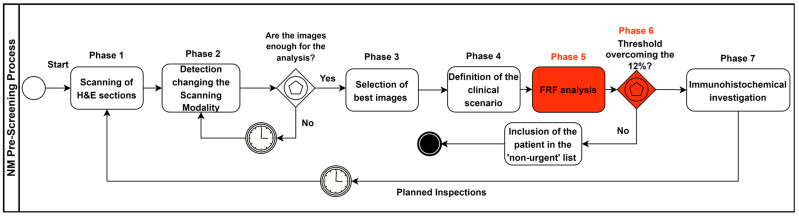
BPMN pre-screening process, including FRF analysis (highlighted in red).

The approach used herein, which is based on FRF image processing, is a part of an innovative clinical protocol based on a pre-screening method able to decrease the health risk/misdiagnosis risk. The clinical approach is mapped via the BPMN scheme shown in Figure 5. 

Such an approach, in our opinion, could be of great assistance in the context of pathology laboratories that have very high volumes of cases and slides, allowing for faster screening of lesions and the initiation of immunohistochemical and, in necessary cases, molecular investigations that can shorten reporting times and improve diagnostic timelines for patients. However, as ML algorithms require a pre-selection by a pathologist as well as a certain amount of data to allow for a high level of reliability, and considering that NM accounts for just under 1% of MM histotypes, it is quite difficult to obtain large amounts of data.

To the best of our knowledge, this is the first paper to address and employ an AI algorithm in pre-screening in dermatopathology.

The very good performance of the algorithm (see the “Precision” parameter, Figure 3) indicates that the algorithm automatically defines the number of instances required for the best computation. A limit is certainly the first setting of the parameter of the algorithm (see the definition of the parameters, such as the Gaussian/Sobel/Hessian filter parameters bilateral/Lipschitz/Gabor/derivative/structured filtering conditions, etc. [13]). Moreover, the high level of sensitivity of the error response was confirmed by slowly varying the algorithm’s parameters. Also, other neural network algorithms could be used for image processing, such as long short-term memory (LSTM). This last algorithm requires the establishment of further settings of parameters calibrated for specific images, which could increase the error calculus. The choice of FRF was then mainly due to the optimisation of the dermatopathology platform to decrease the computational time and simultaneously increase the response accuracy (the main properties of the FRF algorithm). 

It is important to underscore that the indicators adopted for the analysis of the experimental results in Table 5 are:

- The total image area (mm^2^): this indicator provides a reference for the related percentage of the anomalous clusters of the dimension of a specific area according to the image scale (this percentage will increase for a wider image);
- The number of red pixels: the possibly anomalous pixels contained in the image, which represent a primary quantification of the ”risk distribution”;
- Equivalent area (mm^2^): the red pixels are merged to estimate an equivalent area which is useful in defining the final percentage value via losing the information associated with the spatial distribution;
- Equivalent area (%): the final indicator defining the threshold of risk for the pre-screening analysis; the threshold could change after changing the FRF algorithm parameters (a change in the parameters could increase or decrease the number of red pixels, thus changing the threshold value, which is set to 12 % according to the clinical point of view).

## 5. Conclusions

Even today, despite advances in the histopathological diagnosis of atypical, pigmented lesions, NM continues to be a source of diagnostic error. In this paper, we have attempted to introduce an innovative prognostic procedure in dermatopathology to decrease the risk of NM misdiagnosis and to hasten the flow within the pathology laboratory. The procedure was formulated into a preliminary clinical protocol based on the application of the FRF algorithm, which is optimized to be efficient while using a low number of images for training the model. New studies with larger case series are necessary to confirm or otherwise refute our reported results.

## Figures and Tables

**Table 1:** Histological criteria traditionally used in the differential diagnosis of naevoid melanoma and in the supervision process of the algorithm.

Architectural Criteria	Cytological Criteria
Verrucous/warty lesion Dome-shaped lesion	Small, epithelioid, monomorphous cells
Asymmetry Poor circumscription	Round/oval nuclei; small, eosinophilic nucleoli
Possible junctional component	Lack of maturation
Nested growth pattern	Mitoses (in deep)
Variation in nest sizes	Pleomorphism

**Table 2:** Clinical, pathological, histological and immunohistochemical features of the NM analysed in this paper.

Number of Patient	Gender	Age	Topography	Breslow Thickness (mm)	Immunohistochemical Investigations
1	M	74	Back	0.6	Melan-A +−-/HMB-45-
2	M	56	Left shoulder	1.3	Melan-A +−-/HMB-45-
3	M	62	Left leg	2	Melan-A +−-/HMB-45-
4 5	M F	83 47	Right chest Left leg	1.0 3.0	Melan-A +−-/HMB-45- Melan-A +−-/HMB-45-
6	M	43	Back	0.8	Melan-A -/HMB-45-
7	F	46	Back	5	Melan-A +−-/HMB-45-
8	M	60	Right axilla	0.7	Melan-A +−-/HMB-45-
9	F	48	Left flank	1.1	Melan-A-/HMB-45-
10	F	83	Left tibia	0.8	Melan-A ++-/HMB-45-
11	M	49	Back	0.7	Not reported
12	M	39	Abdomen	0.8	Not performed
13	M	43	Left arm	1.0	Melan-A +−-/HMB-45 +--
14	M	44	Not reported	1.2	Not reported
15	M	66	Right scapula	0.8	Melan-A +−-/HMB-45-
16	F	51	Back	0.7	Not reported
17	F	81	Back	0.9	Melan-A ++-/HMB-45-
18	F	91	Left arm	1.1	Not reported

Legend. M: Male; F: female; Melan-A and HMB-45 are reported as semi-quantitative scores.

**Table 3:** Differences in the classification approaches between the methods adopted in [13] and in this work.

Classification	Approach Followed in [13]	Approach Followed in This Work
Number of classes defined in the same image	>2	2 (C_1_: regular clusters; C_2_: possible irregular clusters)
Identification of sure clusters with specified characteristics	Yes	No (the attention is focused on “possible” and uncertain clusters identifying the risk of naevoid melanoma)
Number of images adopted in the entire classification	125 (images with melanoma)	18 (pre-screening images)

**Table 4:** FRF hyperparameters optimizing the training image processing.

Hyperparameter	Value
Filter types	Gaussian blur; Hessian; membrane projections; Sobel; difference of Gaussian
Membrane thickness	1
Membrane patch size	19
Minimum sigma	1
Maximum sigma	16
Total number of classes	2

**Table 5:** Areas of FRF-classified NM pixels in four images from the pool of used images.

Image (1920 × 1088 Pixels)	Total Image Area (mm^2^)	Number of Red Pixels	Equivalent Area (mm^2^) (Probable NM)
NM1	6.66122449	166,689	0.531533801
NM2	13.056	110,438	0.6902375
NM6	3.985226336	140,468	0.267978694
NM8	3.398583923	105,898	0.172288239

**Figure A1:**
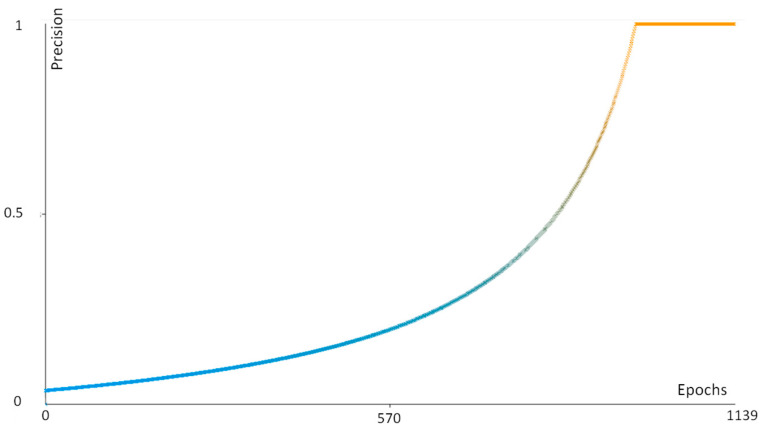
Precision versus epoch number (sample NM8).

**Figure A2:**
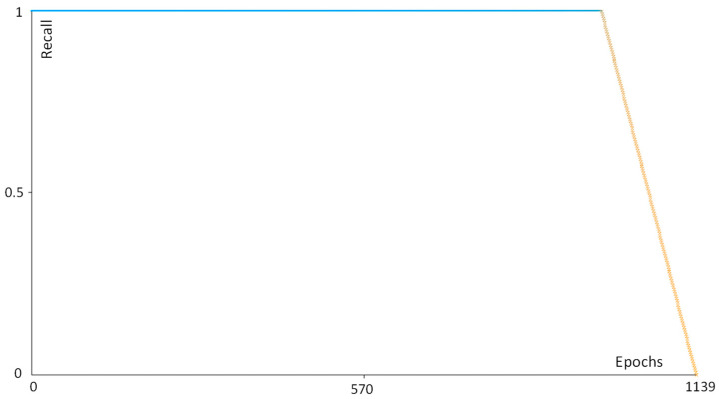
Recall parameter versus epoch number (sample NM8).

**Figure A3:**
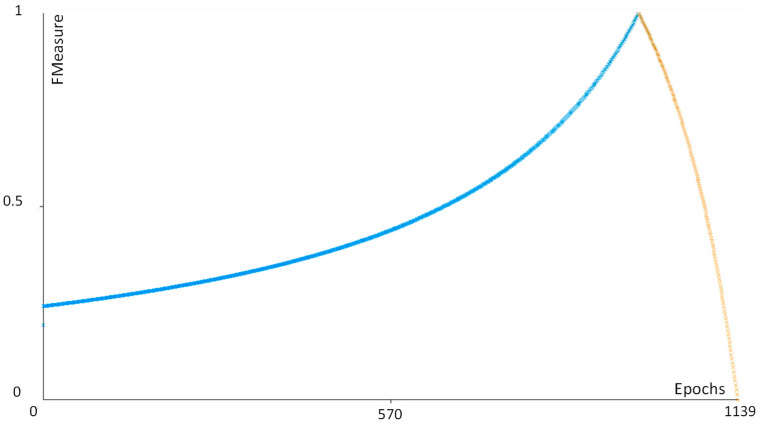
FMeasure parameter versus epoch number (sample NM8).

## Data Availability

Not applicable.

## Appendix A

Table A1 lists the main performance parameters of the adopted FRF algorithm. Figure A1, Figure A2 and Figure A3 show the precision, recall and the Fmeasure parameter plots of the NM8 case, respectively. All the graphs prove the efficiency of the FRF algorithm.

**Table A1:** Main performance parameters of the FRF algorithm (TP: true positive; TN: true negative; FP: false positive; FN: false negative).

Performance Parameter	Function
Precision	TP/(TP + FN)
Recall	TP/(TP + FP)
FMeasure	2TP/(2TP + FP + FN)
